# Instruments for evaluation of motivations for weight loss in individuals with overweight and obesity: A systematic review and narrative synthesis

**DOI:** 10.1371/journal.pone.0220104

**Published:** 2019-07-23

**Authors:** David Franciole Oliveira Silva, Karine Cavalcanti Maurício Sena-Evangelista, Clélia Oliveira Lyra, Lucia Fátima Campos Pedrosa, Ricardo Fernando Arrais, Severina Carla Vieira Cunha Lima

**Affiliations:** 1 Postgraduate Program in Nutrition, Federal University of Rio Grande do Norte, Natal, Rio Grande do Norte, Brazil; 2 Department of Nutrition, Federal University of Rio Grande do Norte, Natal, Rio Grande do Norte, Brazil; 3 Department of Pediatrics, Pediatric Endocrinology Unit, Federal University of Rio Grande do Norte, Natal, Rio Grande do Norte, Brazil; University of Birmingham, UNITED KINGDOM

## Abstract

This systematic review aims to identify instruments used to assess motivations for weight loss in individuals with overweight and obesity from different age groups, such as children, adolescents, adults, and older adults. The virtual search was carried out using the PubMed, Scopus, LILACS, and ADOLEC databases, and by manual search. The following descriptors were used: *questionnaire*, *scale*, *instrument*, *evaluation*, *motivation*, *motive*, *reason*, “*lose weight*,” “*losing weight*,” “*weight loss*,” and *slimming*. Methodological quality was assessed according to the criteria of the COSMIN checklist. The search yielded 3,524 results, seven of which were included in the review. Six questionnaires assessing motivations for weight loss, which could be applied to various age groups, were identified. All the questionnaires presented items related to appearance and health as the main motivation for weight loss. In addition to these motivations, the questionnaires also included items related to improved sports performance, self-confidence, participation in important social events, family and social pressure, and fitting into different clothes. The most evaluated measurement properties in the studies were internal consistency, reliability, content validity, and construct validity. Regarding internal consistency, one was rated as excellent, one as fair, and three as poor. For reliability, two were rated as being of fair quality, and one as of poor quality. Two studies analyzed the content validity and the questionnaires were rated as being of poor methodological quality. Regarding structural validity, one was rated as excellent, another as fair, and another as poor quality. Only the Weight Loss Motivation Questionnaire presented excellent methodological quality for most of the analyzed criteria. There is a need to develop questionnaires that are of better methodological quality to assess motivations for weight loss. Instruments targeting the adolescent population should also be developed.

## Introduction

Obesity is a risk factor for the development of several chronic non-communicable diseases (CNCD), such as cardiovascular disease (CVD), diabetes, and cancer [1–3]. It is considered a public health problem, in addition to increasing the risk of general mortality [4]. According to the World Health Organization (WHO), over 1.9 billion adults over the age of 18 years (39%) are overweight, and 600 million (13%) have obesity [4]. In children and adolescents aged 5 to 17 years, the global prevalence of overweight is 10% and that of obesity is 2 to 3% [5].

In this context, the reduction and control of body weight represent an important measure of prevention and treatment of CNCD [6,7]. However, evidence has shown that often, the main objectives for the reduction of body weight are related to aesthetic value and acceptance by peers [8,9]. When the main motivations for weight loss are for these reasons rather than to obtain a better quality of life and health, the methods used for weight loss are often detrimental to health [10,11]. Among the unhealthy methods most commonly used are fad diets, the use of pills, laxatives, and teas [12,13]. The use of these methods to lose weight may contribute to unsatisfactory results, providing temporary weight loss, and may lead to weight cycling, with weight loss and regain [14].

Success in losing and controlling body weight may be related to the motivation and method chosen to achieve this. Therefore, ascertaining the motivations for weight loss in individuals with overweight and obesity may constitute another tool in the decision-making process for the best strategy for treatment.

Several research tools can be used to assess motivations for weight loss in individuals with overweight and obesity, such as interviews and questionnaires. The use of questionnaires based on adequate methodological criteria may contribute to the validity, reliability, and reproducibility of the results observed in the studies. Identifying the tools for evaluating motivations for weight loss for the different target groups and classifying them according to methodological quality could help to define suitable instruments for use in scientific research.

Hence, given the considerable potential research and clinical implications of identifying the tools for evaluating motivations for weight loss for the different target groups, we conducted a systematic review that aimed to address the overall research question: Which instruments have been used to assess the evaluation of motivation for weight loss in individuals with overweight and obesity and which can be effectively applied to adolescents?

## Methods

### Protocol registration

The systematic review protocol was recorded in PROSPERO (International Prospective Register of Ongoing Systematic Reviews; http://www.crd.york.ac.uk/PROSPERO/ [registration number: CRD42016049039]). Drafting of the manuscript, including the flowchart for screening the records, was performed based on the PRISMA (Preferred Reporting Items for Systematic Reviews and Meta-Analyses) statement [15].

### Inclusion and exclusion criteria

The inclusion criteria used were: 1) publications in Portuguese, English, and/or Spanish; 2) without restriction regarding the period of publication; 3) publications that have elaborated and/or used a questionnaire to assess the motivations for weight loss in individuals with overweight or obesity in any age group; 4) publications that evaluated at least one of the following measurement property: internal consistency, reliability, measurement error, content validity, construct validity (structural validity, hypothesis testing, cross-cultural validity), criterion validity, or responsiveness. Review articles, books, dissertations, and theses were excluded.

### Search strategy

An electronic search was performed by the first author (DS) in the LILACS, PubMed, and Scopus databases, and in the adolescent-specific ADOLEC database to complement the search and to make it more comprehensive in retrieving questionnaires applicable to various age groups. The search strategy is presented in S1 Appendix. The search was carried out on 3 March 2019.

In addition to the search for articles in the databases, unpublished studies were searched in the OpenGrey database (http://www.opengrey.eu/), which provides records of unpublished studies referred to as grey literature. A manual search of the literature was also performed.

### Selection and data extraction

One author (DS) screened the abstracts and full-texts of the search output to identify potentially eligible studies. A second author (SL) checked all articles that the first author decided to exclude after reading the abstract to ensure the screening does not exclude anything that should be included. The second author also evaluated all studies selected for inclusion in the systematic review to ensure selected studies met the inclusion criteria. Contact was made with study authors in the case of possible missing data, which led to clarification of quality assessment for one study. After identifying the studies to be included in the review, two authors (DS and SL) carried out the following data collection procedure: authorship; country in which the study was conducted; language and year of publication; number and age of participants; method of classification of overweight and/or obesity; identification of the questionnaire; items that made up the questionnaire; information on the measurement properties of the questionnaire (internal consistency, reliability, and content and construct validity).

### Methodological quality and level of evidence assessment

Evaluation of the methodological quality of the studies included in the review was performed by two authors (DS and SL) using the COSMIN (Consensus-based Standards for the Selection of Health Measurement Instruments) checklist, which classifies instrument evaluation studies as being of excellent, good, fair, or poor methodological quality [16]. Table 1 presents the definitions of the nine measurement properties assessed.

**Table 1:** Definitions and quality criteria of the measurement properties assessed.

Domain	Measurement property	Definitions and quality criteria
**Reliability**	Internal consistency	“The degree of the interrelatedness among the items” of the questionnaire [17]. Adequate: Cronbach alpha > = 0.70 and < 0.95.
	Reliability	“The proportion of the total variance in the measurements which is because of “true” differences among patients” [17]. Adequate: ICC ≥ 0.70 or Pearson’s r ≥ 0.80.
	Measurement error	“The systematic and random error of a patient’s score that is not attributed to true changes in the construct to be measured” [17]. Adequate: MIC > SDC.
**Validity**	Content Validity	“The degree to which the content of an HR-PRO instrument is an adequate reflection of the construct to be measured” [17]. Adequate: Description of measurement aim, presenting the definition of the construct, relevance to the target population, item selection, and those involved in item selection.
	Construct Validity	
	*Structural Validity*	“The degree to which the scores of an HR-PRO instrument are consistent with hypotheses (for instance with regard to internal relationships, relationships to scores of other instruments, or differences between relevant groups) based on the assumption that the HR-PRO instrument validly measures the construct to be measured” [17]. Adequate: Factor analysis explains ≥ 50% of the total variance.
	*Hypothesis testing*	“The degree to which the scores of an instrument are consistent with hypotheses (for instance with regard to internal relationships, relationships to scores of other instruments, or differences between relevant groups. Based on the assumption that the instrument validly measures the construct to be measured)” [17]. Adequate: Correlation with instruments measuring the same construct (= 0.50) or ≥ 75% of hypotheses conform to expectations.
	*Cross-cultural validity*	“The degree to which the performance of the items on a translated or culturally adapted HR-PRO instrument is an adequate reflection of the performance of the items of the original version of the HR-PRO instrument” [17]. Adequate: Adapted instrument confirms factor structure (or no important differences) of the original instrument.
	Criterion Validity	“The degree to which the scores of an HR-PRO instrument are an adequate reflection of a “gold standard” [17]. Adequate: CC ≥ 0.7 for the gold standard measure.
**Responsiveness**	Responsiveness	“The ability of an HR-PRO instrument to detect change over time in the construct to be measured” [17]. Adequate: Correlation with an instrument measuring the same construct (≥ 0.50) or concordance with hypotheses (≥ 0.70).

CC: Correlation Coefficient; ICC: Intraclass Correlation Coefficient; HR-PRO: Health-Related Patient-Reported Outcomes; MIC: Minimal important change; Pearson’s r = Pearson correlation coefficient; SDC: Smallest Detectable Change.

To evaluate the quality of the studies according to the COSMIN checklist, four steps were followed: identification of the measurement properties used in the studies; identification of the use of statistical methods based on item response theory; identification of responses to all items of the sections belonging to the measurement properties used in the studies; classification of the methodological quality of each property as excellent, good, fair, or poor [16]. The methodological quality of each section corresponds to the lowest rating of any item within the section. For example, if any of the items in the internal consistency section are identified as poor, the methodological quality for internal consistency of the study is classified as poor.

In addition to evaluating the methodological quality of the studies, the overall quality and level of evidence of each measurement property of the questionnaires were also evaluated using the criteria proposed by Terwee et al. [18] and Elbers et al. [19]. Based on these criteria, the overall quality of each property was classified as adequate (+), not adequate (-), or conflicting (±). The criteria are presented in Table 1.

Evidence from studies that evaluated the same questionnaire was synthesized to evaluate the quality of the literature. The level of evidence of each measurement property was determined based on the number of studies, consistency of the results, and quality of evidence.

Thus, the overall quality of a measurement property was classified as having a strong level of evidence when several good quality studies presented the same result (adequate or not adequate) or based on the results of a single excellent quality study. The classification of a moderate level of evidence was given by either several studies of fair quality presenting the same result (adequate or not adequate) or by the outcome of a single good quality study. Evidence was classified as limited when a fair quality study presented the results, as unknown when studies of poor quality presented the same result, and as conflicting when studies presented different results [19].

## Results

**Fig 1:**
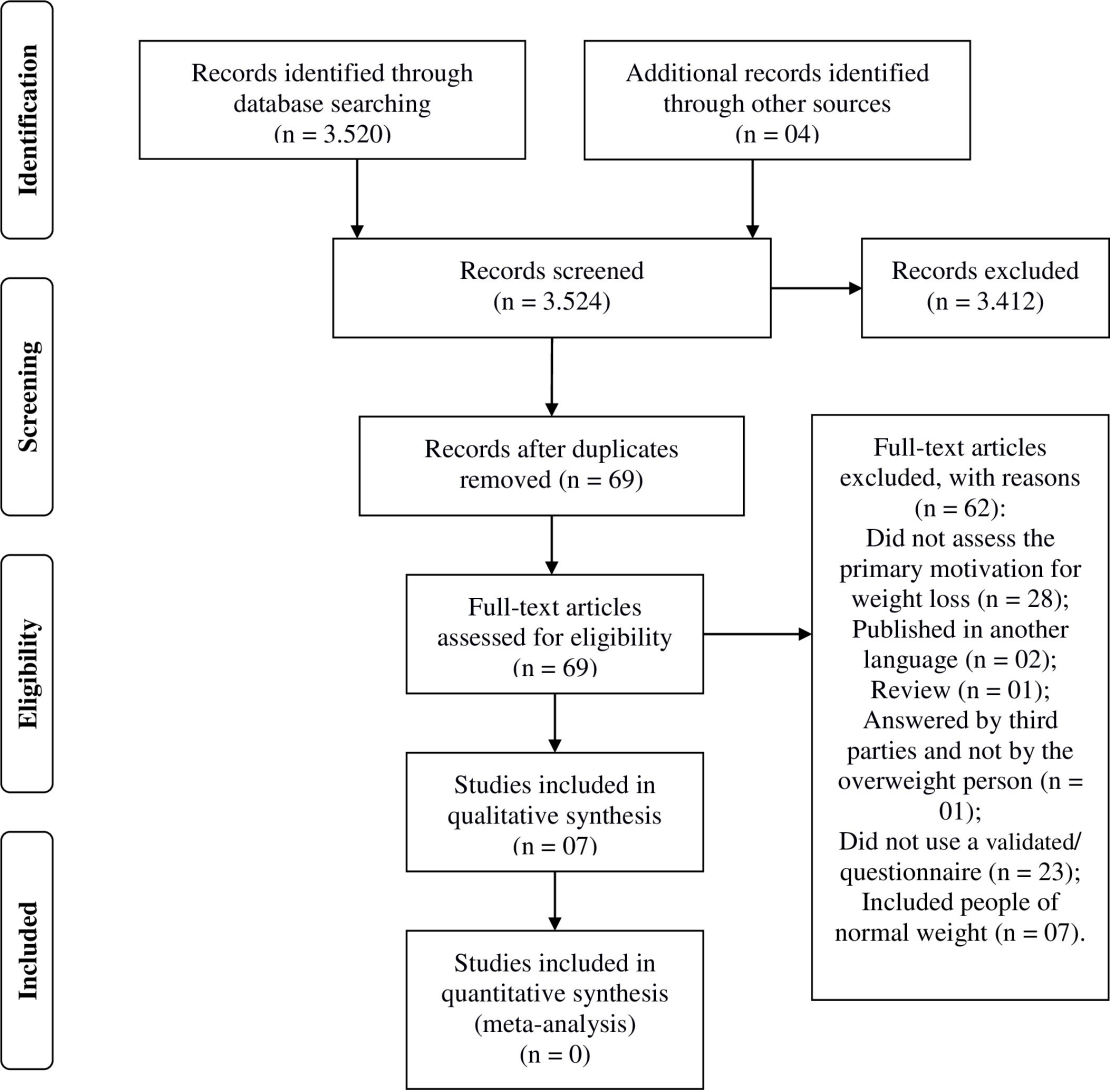
PRISMA flow chart for selection of studies.

The search of the PubMed, Scopus, LILACS, and ADOLEC databases retrieved 3,520 records. In the OpenGrey database, one more record was obtained, and three articles were retrieved using a manual search, totaling 3,524 records. After reading the title and abstract, 3,412 abstracts were excluded. Of the 112 articles selected, 43 were duplicates, with 69 remaining for eligibility assessment. After evaluating the inclusion and exclusion criteria, six questionnaires for the evaluation of motivations for weight loss in individuals with overweight and obesity were included in the review. The questionnaires were: the Braden et al. questionnaire [20], the revised Weight Control Motivation Scale (rWCMS) [21], the Weight Loss Motivation Questionnaire (WLM-Q) [22,23], the Motivation for Weight Loss Questionnaire (MWLQ) [24], the Primary Goals for Weight Loss Questionnaire (PGWLQ) [25], and the Rancourt et al. questionnaire [26]. Fig 1 shows the flowchart for the selection of the studies; the list of excluded studies along with reasons for exclusion is presented in S2 Appendix.

### Study characteristics

The characteristics of the studies included in the review are presented in Table 2. The sample size of each study ranged from 49 [26] to 6,007 subjects [21], and the age ranged from eight [20] to 74 years [22,23]. Three studies were conducted in the United States of America [20,24,26], two in Switzerland [22,23], one in Canada [21], and one in Australia [25].

**Table 2:** Characteristics of the studies included in the review.

Author (Year)	Country	N	Age group (years)	Diagnostic criteria of overweight and/or obesity	Number of domains/items of the questionnaire
**Braden et al. [20]**	United States	77	8 to 12	WHO (>85th Percentile)	2/10
**Stotland et al. [21]**	Canada	6.007	19 to 65	WHO (BMI ≥ 25 kg/m²)	2/8
**Meyer et al. [22]**	Switzerland	355	15 to 74	WHO (BMI ≥ 25 kg/m²)	3/24
**Schelling et al. [23]**	Switzerland	302	15 to 74	WHO (BMI≥ 25 kg/m²)	3/24
**Ames et al. [24]**	United States	67 women	18 to 30	BMI > 28 and< 40 kg/m²	5/27
**Murphy et al. [25]**	Australia	127 women	23 to 71	WHO (BMI ≥ 25 kg/m²)	9/87
**Rancourt et al. [26]**	United States	49	14 to 20	Center for Disease Control (BMI z-scores)	3/12

BMI: Body mass index; WHO: World Health Organization.

### Weight loss motivation questionnaires

The number of domains ranged from two [20,21] to nine [25]. The most frequent names given to domains were motives related to appearance and health, present in four studies [22–25].

The number of items in the questionnaires ranged from eight [21] to 87 [25]. Five questionnaires contained items related to appearance and health as the main motivations for weight loss [21–25]. In addition to these motivations, questionnaires also included items related to improved performance in sports [20,25], self-confidence [21–25], participation in an important social event [25], family pressure [20], social pressure [22–25], fitting into different clothes [20,22–25], climbing up and down stairs more easily, improving sleep, reducing leg pain, avoiding surgery, and improving sexual performance [25].

### Methodological quality and level of evidence

The measurement properties used and the methodological quality of the studies are described in Tables 3 and 4. Five studies evaluated the internal consistency of the questionnaire using Cronbach’s alpha [21,22,24,25,26]. According to the COSMIN checklist, in terms of methodological quality, one can be classified as excellent [22], one as fair [21], and three as poor [24,25,26]. As for reliability, of the three studies evaluated [22,24,25], two were classified as of fair quality [22,25], and one was of poor quality [24].

**Table 3:** Psychometric characteristics of the questionnaires included in the systematic review.

Measurement property	Braden et al [20]	Stotland et al. [21]/ rWCMS	Meyer et al. [22]/ WLM-Q	Schelling et al. [23]/ WLM-Q	Ames et al. [24]/ MWLQ	Murphy et al. [25]/ PGWLQ	Rancourt et al. [26]
**Internal consistency**	Not evaluated	CA: 0.68–0.79 for the five subscales	CA: 0.93 for all 24 items	Data not presented	CA: 0.62–0.86.	CA: 0.74–0.91 for the nine subscales	CA: 0.76–0.86 for the three subscales
**Reliability**	Not evaluated	Not evaluated	TR (ICC): 0.74–0.82 over 1 week	Data not presented	TR (*r)*: 0.95 over 1 week	TR (*r*): 0.30–0.55 over time 1 and 2, not presented	Not evaluated
**Measurement error**	Not evaluated	Not evaluated	Not evaluated	Not evaluated	Not evaluated	Not evaluated	Not evaluated
**Content Validity**	The theoretical foundation of the construct and item selection and/or those involved in item selection was not clearly described	Not evaluated	Not evaluated	Not evaluated	Not evaluated	The theoretical foundation of the construct and item selection and/or those involved in item selection was not clearly described	Not evaluated
***Structural Validity***	Not evaluated	> 60% of the total variance explained (factor analysis).	>53% of the total variance explained (factor analysis)	Data not presented	Not evaluated	Physical appearance (>61%) and psychological aspect: (>71%) of the total variance explained (factor analysis).	Not evaluated
***Hypothesis testing***	Not evaluated	Not evaluated	Not evaluated	Not evaluated	Not evaluated	Convergent: PGWLQ factors and the MWLQ subscales (CC): the most similar factors/ subscales < 0.50.	Not evaluated
**Cross-cultural validity**	Not evaluated	Not evaluated	Not evaluated	Not evaluated	Not evaluated	Not evaluated	Not evaluated
**Criterion Validity**	Not evaluated	Not evaluated	Not evaluated	Not evaluated	Not evaluated	Not evaluated	Not evaluated
**Responsiveness**	Not evaluated	Not evaluated	Not evaluated	Not evaluated	Not evaluated	Not evaluated	Not evaluated

CA: Cronbach’s alpha; CC: Correlation Coefficients; ICC: Intraclass Correlation Coefficients; CRC: Corresponding Repeatability Coefficients; MWLQ–Motivation for Weight Loss Questionnaire; r = Pearson’s r; PGWLQ–Primary Goals for Weight Loss Questionnaire; rWCMS–Revised Weight Control Motivation Scale; TR: Test-retest; WLMQ–Weight Loss Motivation Questionnaire.

**Table 4:** Methodological quality of the studies included in the systematic review, according to the criteria of the COSMIN checklist.

Measurement property	Braden et al. [20]	Stotland et al. [21]/ rWCMS	Meyer et al.[22]/ WLM-Q	Schelling et al.[23] /WLM-Q	Ames et al.[24]/ MWLQ	Murphy et al.[25]/ PGWLQ	Rancourt et al.[26]
**Internal consistency**	-	Fair	Excellent	-	Poor	Poor	Poor
**Reliability**	-	-	Fair	-	Poor	Fair	-
**Measurement error**	-	-	-	-	-	-	-
**Content Validity**	Poor	-	-	-	-	Poor	-
**Structural Validity**	-	Fair	Excellent	-	-	Poor	-
**Hypothesis testing-**	-	-	-	-	-	Poor	-
**Cross-cultural validity**	-	-	-	-	-	-	-
**Criterion Validity**	-	-	-	-	-	-	-
**Responsiveness**	-	-	-	-	-	-	-

rWCMS–Revised Weight Control Motivation Scale; WLM-Q–Weight Loss Motivation Questionnaire; MWLQ–Motivation for Weight Loss Questionnaire; PGWLQ–Primary Goals for Weight Loss Questionnaire.

Two studies carried out an analysis of content validity and were classified according to the criteria of the COSMIN checklist as being of poor methodological quality [20,25]. Three studies analyzed the structural validity of the questionnaire by factor analysis [21,22,25]. One was classified as of excellent quality [22], another as of fair quality [21], and another as of poor methodological quality regarding structural validity [25]. The study that evaluated hypothesis testing was methodologically poor because of a lack of information on the measurement properties of the comparator instrument [25]. None of the seven studies reported evaluation of measurement error, cross-cultural validity, criterion validity, and responsiveness.

The overall quality and level of evidence for each measurement property are presented in Table 5. For internal consistency, the rWCMS [21] presented inadequate overall quality and a limited level of evidence, the WLM-Q [22,23] presented adequate overall quality and a strong level of evidence, the MWLQ [24] presented inadequate overall quality and an unknown level of evidence, the PGWLQ [25] presented an adequate overall quality and an unknown level of evidence, and the Rancourt et al. questionnaire [26] presented adequate overall quality and an unknown level of evidence. For reliability, the rWCMS [21] was classified as having inadequate overall quality and a limited level of evidence, the WLM-Q [22,23] presented adequate overall quality and a limited level of evidence, the MWLQ [24] presented an adequate overall quality and an unknown level of evidence, and the PGWLQ [25] presented an inadequate overall quality and limited evidence. The Braden et al. questionnaire [20] and the PGWLQ [25] questionnaires were classified for content validity as having inadequate overall quality and an unknown level of evidence. Construct validity of the rWCMS questionnaire [21] was classified as adequate with limited evidence, the WLM-Q [22,23] presented adequate overall quality with a strong level of evidence, and the PGWLQ [25] presented adequate overall quality with an unknown level of evidence. The PGWLQ questionnaire was classified for hypothesis testing as having inadequate overall quality and an unknown level of evidence [25].

**Table 5:** Overall quality and level of evidence of the measurement properties of the questionnaires included in the systematic review.

Measurement property	Braden *et al*. [20]	rWCMS [21]	WLM-Q [22,23]	MWLQ [24]	PGWLQ [25]	Rancourt *et al*. [26]
**Internal consistency**	-	Not adequate (Limited)	Adequate (Strong)	Not adequate (Unknown)	Adequate (Unknown)	Adequate (Unknown)
**Reliability**	-	-	Adequate (Limited)	Adequate (Unknown)	Not adequate (Limited)	-
**Measurement error**	-	-	-	-	-	-
**Content Validity**	Not adequate (Unknown)	-	-	-	Not adequate (Unknown)	-
**Structural Validity**	-	Adequate (Limited)	Adequate (Strong)	-	Adequate (Unknown)	-
**Hypothesis testing**	-	-	-	-	Not adequate (Unknown)	-
**Cross-cultural validity**	-	-	-	-	-	-
**Criterion Validity**	-	-	-	-	-	-
**Responsiveness**	-	-	-	-	-	-

rWCMS–Revised Weight Control Motivation Scale; WLM-Q–Weight Loss Motivation Questionnaire; MWLQ–Motivation for Weight Loss Questionnaire; PGWLQ–Primary Goals for Weight Loss Questionnaire.

## Discussion

The methodological quality of most studies on weight loss motivation assessment questionnaires included in this review is fair or poor [20,21,24–26], according to the COSMIN checklist criteria [27], because of several methodological limitations in the internal consistency, reliability, hypothesis testing, and content and construct validity. The Weight Loss Motivation Questionnaire was the only questionnaire of excellent methodological quality for most of the criteria analyzed [22,23].

The analysis of the measurement properties of the most used questionnaires in the studies was the evaluation of internal consistency, by Cronbach’s alpha, which reflects the level of correlation between the items of an instrument resulting from the application to a significant sample of subjects. The WLM-Q [22], the PGWLQ [25], and the Rancourt et al. questionnaire [26] presented adequate internal consistency, evidenced by Cronbach’s alpha values between 0.70 and 0.95. However, although the methodological quality of the study using the WLM-Q [22] was excellent and the level of evidence for this was strong, for the PGWLQ and the Rancourt et al. questionnaire [25,26], the level of evidence was unknown because of the poor methodological quality of the study.

As for the reliability assessment, which represents the ability to reproduce a result consistently in time and/or space, or with different observers, a higher value of test-retest was observed in the MWLQ [24], with test-retest reliability of *r* = 0.95, over a period of one week, presenting adequate quality. However, it is emphasized that the use of Pearson’s and Spearman’s correlation coefficient is not considered adequate because it does not take systematic error into account.

Validity refers to whether the instrument measures exactly what it proposes to measure. The questionnaire by Braden et al. [20] and the PGWLQ [25] presented inadequate content validity because of the theoretical foundation of motivations for weight loss, the purpose of the instrument, the process of item selection, and/or those involved in item selection not being clearly described or defined.

The rWCMS [21], WLM-Q [22], and PGWLQ [25] questionnaires showed adequate structural validity since the confirmatory factorial analysis explained more than 50% of the total variance. It should be emphasized that other measures such as criterion validity and hypothesis testing (convergent and divergent validity), not used in most of the studies included in the review, are necessary to infer about the adequacy of the construct validity of an instrument.

There is no gold-standard comparator in the validation of questionnaires to evaluate the motivations for weight loss in individuals with overweight and obesity. In this context, the PGWLQ [25] presented inadequate hypothesis testing because of the lack of information on the measurement properties of the comparator instrument, the MWLQ, as well as the values of correlation coefficients, which were <0.5 for most of the similar factors/subscales.

The instruments used to assess motivations for weight loss in individuals with overweight and obesity were designed for distinct target groups, including children [20], adolescents [22,23,26], adults [21–25], and older adults [21–25]. However, even though three questionnaires were applied to adolescents [22,23,26], it should be emphasized that there is a need for a validated questionnaire specifically directed at the evaluation of motivations for weight loss in adolescents with overweight or obesity (10 to 19 years). This would better capture specific motivations for weight loss that may exist in this group such as bullying [27–29], the desire to be more popular in school/peer acceptance [28,30], and celebrating the fifteenth birthday [27], which are not included in the existing questionnaires.

The questionnaires for evaluating motivations for weight loss identified in this study have in common the inclusion of items related to appearance and health, which represented the main motivations for weight loss [20–25]. The motivations for weight loss in individuals with overweight and obesity are influenced by several factors such as sex, age, and health status. Motivations for weight loss related to health and fitness are more common in men, while women are more prone to motivations related to appearance and being able to fit into clothes [8,20,21]. A study with 248 Americans (50.8% women) older than 18 years and with a BMI ≥ 25 kg/m^2^, found that 80.2% of the women vs. 58.2% of the men (p <0.05) presented a motivation related to improving the appearance [31].

Regarding age, appearance-based motivation is more frequent among younger people, whereas motivation related to health is more frequent in older than in younger individuals. Kalarchian et al. [32], evaluating 203 women aged 18–55 years with BMI > 27 and < 40 kg/m^2^, observed differences in the mean age between appearance versus health motivated individuals for weight loss (appearance: 38.9 vs. health: 41.6 years). A study with 1,785 adults with obesity (1,393 women and 392 men; median age: 46 years) also found a greater tendency for appearance-based motivations among the youngest individuals (appearance: 38.2 vs. present health: 47.3 years, p<0.001) [33].

Metabolic complications related to obesity, such as insulin resistance, dyslipidemia, and hypertension frequently require greater attention to health and the need to search for health care [34]. In this context, patients with higher BMI and/or metabolic complications related to obesity also present a greater tendency to present health-based motivation, while those with lower BMI are more likely to be motivated by appearance [8,21]. Dalle Grave et al. [33] observed that women seeking treatment to improve appearance had a lower grade of obesity (appearance: 36.9 kg/m^2^ vs. present health: 38.8 kg/m^2^). A study with 526 Kuwaiti adults (mean BMI = 34.2 kg/m^2^) identified that 19.5% of individuals with morbid obesity attempted to lose weight for reasons of personal appearance compared to 32.4% of individuals with overweight [35].

Besides motivations related to appearance and health, family and social factors such as participation in an important social event and the pressure from parents and/or friends are motivations that are also analyzed in most of the questionnaires included in the present study [20,22–24]. These motivations refer to the goals of satisfying the desires of third parties, in order to achieve social acceptance, with the consideration that individuals with such motivations from family and peer pressure may have low self-esteem.

It should be emphasized that the results of studies using validated questionnaires, such as those included in this review, were different from those that did not use validated questionnaires. Dalle Grave et al. [36] evaluated the motivations for weight loss in 1,000 individuals with overweight using an unstructured and non-validated questionnaire, finding that motivations for weight loss related to appearance among women was 20.5% and 8.5% among men. These results differ from those found in the studies included in this review, in which the motivation for appearance-related weight loss is reported by 80% of participants in a study by Braden et al. [20].

In addition to the instruments evaluated in the present systematic review and the focus on reasons related to health, appearance, and social factors, there are instruments that evaluate the autonomous motivation of individuals in relation to engagement for weight loss [37]. Among these instruments is the Autonomous Motivation subscale of the Treatment Self-Regulation Questionnaire (TSRQ) [37], derived from self-regulation theory. Williams et al. (1996) [37] verified that individuals with obesity engaged in a very low-calorie diet that presents autonomous motivation, measured by TSRQ, experienced successful weight loss.

This systematic review found that the overall quality of the literature supporting the psychometric properties of instruments for evaluation of motivations for weight loss in individuals with overweight and obesity is low. Given the methodological limitations identified in the present study, it is recommended that further research is carried out to develop and validate psychometrically sound instruments.

The strengths of this systematic review include the originality of the study, the evaluation of the methodological quality of the studies, and the evaluation of the overall quality and level of evidence of each measurement property. Among the limitations of this study is the possibility of publication bias because studies with negative results are less likely to be published. However, the effect of publication bias may have been less important in this study than in other systematic reviews, considering that were validated tools to measure motivation for weight loss, not the efficacy of treatments. Another limitation, with respect to publication bias, concerns the exclusion of studies that were not published in Portuguese, English, or Spanish. However, majority of the most renowned journals are typically published in English. In order to minimize the effects of publication bias, searches of the main health databases, grey literature, and a manual search were conducted. The screening of the abstracts and full-texts to identify potentially eligible studies by one author is another potential limitation. However, a second author also checked all articles that the first author decided to exclude after reading the abstract to ensure the screening does not exclude anything that should be included. In addition, the second author also evaluated all studies selected for inclusion in the systematic review to ensure that the selected studies met the inclusion criteria. The use of a standard datasheet for extracting the data, as well as the extraction of the data being performed by two researchers, contributed to minimizing the potential bias in the assessment of studies included.

## Conclusions

In the present review, six weight loss motivation questionnaires were identified for individuals with overweight and obesity, applicable to several age groups. The number of domains and items differed among the questionnaires, but all presented items related to motivations for weight loss due to appearance and health. The most evaluated measurement properties in the studies were internal consistency, reliability, content validity, and construct validity. Only the Weight Loss Motivation Questionnaire presented excellent methodological quality for most of the analyzed criteria. There is a need to develop questionnaires of better methodological quality to assess motivations for weight loss, as well as to develop an instrument directed at the adolescent population.

## Supporting information

S1 Checklist PRISMA 2009 checklist. (DOCX)

S1 Appendix Full electronic search strategy for PubMed, Scopus, LILACS, and ADOLEC databases. (DOCX)

S2 Appendix List of excluded studies along with reasons for exclusion. (DOCX)
