# Supplementary material for: Instruments for evaluation of motivations for weight loss in individuals with overweight and obesity: A systematic review and narrative synthesis
Source: PLoS One. 2019 Jul 23;14(7):e0220104. doi: 10.1371/journal.pone.0220104 (PMC6650081; doi:10.1371/journal.pone.0220104)
Supplement: S1 Appendix — (DOCX) [file pone.0220104.s002.docx]

# S1 Appendix. Full electronic search strategy for PubMed, Scopus, LILACS, and ADOLEC databases

**PubMed**

(questionnaire OR scale OR instrument OR evaluation) AND (motivation OR motivations OR “motivating factor” OR "motivating factors" OR motive OR motives OR reason OR reasons OR predictor) AND (“lose weight "OR" losing weight" OR "weight loss" OR slimming)

**Scopus**

TITLE-ABS-KEY (questionnaire OR scale OR instrument OR evaluation) AND TITLE-ABS-KEY (motivation OR motivations OR "motivating factor" OR “motivating factors” OR motive OR motives OR reason OR reasons OR predictor) AND TITLE-ABS-KEY ("lose weight" OR “losing weight” OR "weight loss" OR "slimming")

**Limitations:** Portuguese OR English OR Spanish

**LILACS**

(questionário OR escala OR instrumento OR avaliação) AND (motivação OR motivações OR motivo OR motivos OR razão OR razões) AND (“perda de peso” OR “perder peso” OR emagrecimento OR emagrecer)

**ADOLEC**

(questionário OR escala OR instrumento OR avaliação) AND (motivação OR motivações OR motivo OR motivos OR razão OR razões) AND (“perda de peso” OR “perder peso” OR emagrecimento OR emagrecer)

The search was commenced in September 2017 and the last update was performed on March 3rd 2019.
