# Supplementary material for: Instruments for evaluation of motivations for weight loss in individuals with overweight and obesity: A systematic review and narrative synthesis
Source: PLoS One. 2019 Jul 23;14(7):e0220104. doi: 10.1371/journal.pone.0220104 (PMC6650081; doi:10.1371/journal.pone.0220104)
Supplement: S2 Appendix — (DOCX) [file pone.0220104.s003.docx]

**S2 Appendix. List of excluded studies along with reasons for exclusion.**

**Full-text articles excluded, with reasons (n=62)**

| **Study** | **Reason for Exclusion** |
| --- | --- |
| Hirschfeld‐Dicker et al., 2019 [1] | Included people of normal weight |
| Rafiei N and Gill, 2018 [2] | Did not use a validated questionnaire |
| Hansen et al., 2018 [3] | Did not assess the primary motivation for weight loss |
| Vörös et al., 2018 [4] | Included people of normal weight |
| Halali et al., 2018 [5] | Included people of normal weight |
| Elsborg and Elbe, 2018 [6] | Did not assess the primary motivation for weight loss |
| Batch et al., 2018 [7] | Did not assess the primary motivation for weight loss |
| Sasdelli et al., 2018 [8] | Did not use a validated questionnaire |
| Saez et al., 2018 [9] | Did not assess the primary motivation for weight loss |
| Coffey et al., 2018 [10] | Included people of normal weight |
| Czeglédi, 2017 [11] | Article published in another language other than English or Portuguese or Spanish |
| Bauer et al., 2017 [12] | Did not assess the primary motivation for weight loss |
| Mazloomy-Mahmoodabad et al., 2017 [13] | Did not assess the primary motivation for weight loss |
| Fonvig et al., 2017 [14] | Did not assess the primary motivation for weight loss |
| Vincze et al., 2017 [15] | Did not use a validated questionnaire |
| Maclin-Akinyemi et al., 2017 [16] | Did not use a validated questionnaire |
| Zizzi et al., 2016 [17] | Did not assess the primary motivation for weight loss |
| Smalley et al., 2016 [18] | Did not assess the primary motivation for weight loss |
| Brown et al., 2016 [19] | Did not use a validated questionnaire |
| Tigay et al., 2016 [20] | Did not assess the primary motivation for weight loss |
| Lous et al., 2016 [21] | Did not use a validated questionnaire |
| Katz et al., 2015 [22] | Did not assess the primary motivation for weight loss |
| Ceccarini et al., 2015 [23] | Review |
| Leão et al., 2015 [24] | Did not assess the primary motivation for weight loss |
| Chang and Chiou, 2015 [25] | Did not use a validated questionnaire |
| Robertson et al., 2014 [26] | Did not use a validated questionnaire |
| Armitage et al., 2014 [27] | Did not assess the primary motivation for weight loss |
| Tan and Wong, 2014 [28] | Did not use a validated questionnaire |
| Epperson et al., 2014 [29] | Did not use a validated questionnaire |
| Siervo et al., 2014 [30] | Did not assess the primary motivation for weight loss |
| Puhl et al., 2013 [31] | Did not assess the primary motivation for weight loss |
| Yoong et al., 2013 [32] | Did not use a validated questionnaire |
| LaRose et al., 2013 [33] | Included people of normal weight |
| Woolford et al., 2012 [34] | Answered by third parties and not by the individual with overweight |
| Calugi et al., 2012 [35] | Did not use a validated questionnaire |
| Puhl et al., 2011 [36] | Did not assess the primary motivation for weight loss |
| Kalarchian et al., 2011 [37] | Did not use a validated questionnaire |
| Cresci et al., 2011 [38] | Did not assess the primary motivation for weight loss |
| Edmiston and Wagner, 2010 [39] | Did not assess the primary motivation for weight loss |
| Jay et al., 2010 [40] | Did not assess the primary motivation for weight loss |
| Elfhag and Rössner, 2010 [41] | Did not assess the primary motivation for weight loss |
| Dalle Grave et al., 2009 [42] | Did not assess the primary motivation for weight loss |
| Cresci and Rotella, 2009 [43] | Did not assess the primary motivation for weight loss |
| Kwan, 2009 [44] | Did not use a validated questionnaire |
| Chałabis and Lange, 2008 [45] | Article published in another language other than English or Portuguese or Spanish |
| Johannessen and Berntsen, 2009 [46] | Did not assess the primary motivation for weight loss |
| Alm et al., 2008 [47] | Did not use a validated questionnaire |
| Heading, 2008 [48] | Did not use a validated questionnaire |
| Annunziato and Lowe, 2007 [49] | Did not assess the primary motivation for weight loss |
| Georgiadis et al., 2006 [50] | Did not assess the primary motivation for weight loss |
| Murtagh et al., 2006 [51] | Did not use a validated questionnaire |
| Dalle Grave et al., 2005 [52] | Did not use a validated questionnaire |
| Wannamethee et al., 2005 [53] | Did not assess the primary motivation for weight loss |
| Dalle Grave et al., 2004 [54] | Did not use a validated questionnaire |
| Putterman and Linden, 2004 [55] | Included people of normal weight |
| Hawks et al., 2003 [56] | Did not assess the primary motivation for weight loss |
| Clarke, 2002 [57] | Did not use a validated questionnaire |
| Reeder et al., 1997 [58] | Included people of normal weight |
| Tinker and Tucker, 1997 [59] | Did not use a validated questionnaire |
| Fontaine et al., 1997 [60] | Did not use a validated questionnaire |
| Striegel‐Moore et al., 1996 [61] | Did not use a validated questionnaire |
| Harris et al., 1990 [62] | Did not assess the primary motivation for weight loss |

**References:**

1. Hirschfeld‐Dicker L, Samuel RD, Tiram Vakrat E, Dubnov‐Raz G. Preferred weight‐related terminology by parents of children with obesity. Acta Paediatrica. 2018;108(4):712-17.

2. Rafiei N, Gill T. Identification of factors contributing to successful self‐directed weight loss: a qualitative study. J Hum Nutr Diet. 2018;31(3):329-336.

3. Hansen S, Huttunen-Lenz M, Sluik D, Brand-Miller J, Drummen M, Fogelholm M, et al. Demographic and Social-Cognitive Factors Associated with Weight Loss in Overweight, Pre-diabetic Participants of the PREVIEW Study. Int J Behav Med. 2018; 25(6):682-692.

4. Vörös K, Márkus B, Hargittay C, Sisak O, Kalabay L. Perceived cardiovascular risk and attitude towards lifestyle change. Fam Pract. 2018;35(6):712-717.

5. Halali F, Lapveteläinen A, Karhunen L, Saarela AM, Lappalainen R, Kantanen T. Motivators, barriers and strategies of weight management: A cross-sectional study among Finnish adults. Eat Behav. 2018;31:80-87.

6. Elsborg P, Elbe AM. Exercise-specific volition and motivation for weight loss maintenance following an intensive lifestyle intervention. Health Psychol. 2018;37(8):759.

7. Batch BC, Goldstein K, Yancy Jr WS, Sanders LL, Danus S, Grambow SC, Bosworth HB. Outcome by gender in the veterans health administration motivating overweight/obese veterans everywhere weight management program. J Womens Health. 2018;27(1):32-39.

8. Sasdelli AS, Petroni ML, Paoli AD, Collini G, Calugi S, Dalle Grave R, Marchesini G. Expected benefits and motivation to weight loss in relation to treatment outcomes in group-based cognitive-behavior therapy of obesity. Eat Weight Disord. 2018;23(2):205-214.

9. Saez L, Legrand K, Alleyrat C, Ramisasoa S, Langlois J, Muller L, Briançon S. Using facilitator–receiver peer dyads matched according to socioeconomic status to promote behaviour change in overweight adolescents: a feasibility study. BMJ Open. 2018;8(6):e019731.

10. Coffey F, Curran TI, Kenny C, Holmes K. Assessment of patients’ attitudes towards weight loss in an Irish general practice setting. Ir J Med Sci. 2018;1971:1-6.

11. Czeglédi E. Motivation for weight loss among weight loss treatment participants. Orv Hetil. 2017;158(49):1960-1967.

12. Bauer AG, Berkley-Patton J, Bowe-Thompson C, Ruhland-Petty T, Berman M, Lister S, Christensen K. Do Black Women's Religious Beliefs About Body Image Influence Their Confidence in Their Ability to Lose Weight?. Prev Chronic Dis. 2017;14:E98-E98.

13. Mazloomy-Mahmoodabad SS, Navabi ZS, Ahmadi A, Askarishahi M. The effect of educational intervention on weight loss in adolescents with overweight and obesity: Application of the theory of planned behavior. ARYA atherosclerosis. 2017;13(4):176.

14. Fonvig CE, Hamann SA, Nielsen TRH, Johansen MØ, Grønbæk HN, Mollerup PM, Holm JC. Subjective evaluation of psychosocial well-being in children and youths with overweight or obesity: the impact of multidisciplinary obesity treatment. Qual Life Res. 2017;26(12):3279-3288.

15. Vincze L, Rollo ME, Hutchesson MJ, Burrows TL, MacDonald-Wicks L, Blumfield M, Collins CE. A cross sectional study investigating weight management motivations, methods and perceived healthy eating and physical activity influences in women up to five years following childbirth. Midwifery. 2017;49:124-133.

16. Maclin-Akinyemi C, Krukowski RA, Kocak M, Talcott GW, Beauvais A, Klesges RC. Motivations for weight loss among active duty military personnel. Mil Med. 2017;182(9-10):e1816-e1823.

17. Zizzi SJ, Lima Fogaca J, Sheehy T, Welsh M, Abildso C. Changes in weight loss, health behaviors, and intentions among 400 participants who dropped out from an insurance-sponsored, community-based weight management program. J Obes. 2016;2016:10p.

18. Smalley KB, Warren JC, McClendon S, Peacock W, Caro M. Ethnic Identity Attachment and Motivation for Weight Loss and Exercise among Rural, Overweight, African-American Women: Supplementary Issue: Health Disparities in Women. Clin Med Insights Womens Health. 2016;9:CMWH-S34691.

19. Brown CL, Skelton JA, Perrin EM, Skinner AC. Behaviors and motivations for weight loss in children and adolescents. Obesity. 2016;24(2):446-452.

20. Tigay JH, Thompson M, Sutton D, Lesley M. Motivation for Weight Loss. J Nurse Pract. 2016;12(4):e169-e173.

21. Lous J, Freund KS Predictors of weight loss in young adults who are over-weight or obese and have psychosocial problems: a post hoc analysis. BMC Fam Pract. 2016;17(1):43.

22. Katz I, Madjar N, Harari A. Parental support and adolescent motivation for dieting: The self-determination theory perspective. J Psychol. 2015;149(5):461-479.

23. Ceccarini M, Borrello M, Pietrabissa G, Manzoni GM, Castelnuovo G. Assessing motivation and readiness to change for weight management and control: an in-depth evaluation of three sets of instruments. Front Psychol. 2015;6:511.

24. Leão JM, Lisboa LCV, Pereira MDA, Lima LDF, Lacerda KC, Elias MAR, et al. Motivational stages to behavioral change in beginners of weight loss treatment. J Bras Psiquiatr. 2015;64(2):107-114.

25. Chang YYC, Chiou WB. Means yield to ends in weight loss: Focusing on “how” vs “why” aspects of losing weight can lead to poorer regulation of dietary practices. J Acad Nutr Diet. 2015;115(9):1387-1391.

26. Robertson A, Mullan B, Todd J. A qualitative exploration of experiences of overweight young and older adults. An application of the integrated behaviour model. Appetite. 2014;75:157-164.

27. Armitage CJ, Wright CL, Parfitt G, Pegington M, Donnelly LS, Harvie MN. Self-efficacy for temptations is a better predictor of weight loss than motivation and global self-efficacy: Evidence from two prospective studies among overweight/obese women at high risk of breast cancer. Patient Educ Couns. 2014;95(2):254-258.

28. Tan WJM, Wong TKM. Demographic profile, clinical characteristics, motivations and weight loss outcomes of patients in a nonsurgical weight management programme. Singapore Med J. 2014;55(3):150.

29. Epperson AE, Song AV, Wallander JL, Markham C, Cuccaro P, Elliott MN, Schuster MA. Associations among body size, body image perceptions, and weight loss attempts among African American, Latino, and White youth: A test of a mediational model. J Pediatr Psychol. 2014.39(4):394-404.

30. Siervo M, Montagnese C, Muscariello E, Evans E, Stephan BCM, Nasti G, et al. Weight loss expectations and body dissatisfaction in young women attempting to lose weight. J Hum Nutr Diet. 2014;27:84-89.

31. Puhl R, Peterson JL, Luedicke J. Motivating or stigmatizing? Public perceptions of weight-related language used by health providers. Int J Obes. 2013;37(4):612.

32. Yoong SL, Carey ML, Sanson-Fisher RW, D’Este CA. A cross-sectional study assessing Australian general practice patients’ intention, reasons and preferences for assistance with losing weight. BMC Fam Pract. 2013;14(1):187.

33. LaRose JG, Leahey TM, Hill JO, Wing RR. Differences in motivations and weight loss behaviors in young adults and older adults in the National Weight Control Registry. Obesity. 2013;21(3):449-453.

34. Woolford SJ, Sallinen BJ, Clark SJ, IglayReger HB, Gordon PM. Association Between Physician Recommendation for Adolescents to Join a Weight Loss Program and BMI Change. J Prim Care Community Health. 2012;3(2):83-87.

35. Calugi S, Dalle Grave R, Compare A, Dall’Aglio E, Petroni ML, Marchesini G, QUOVADIS Study Group. Weight loss and clinical characteristics of young adults patients seeking treatment at medical centers: Data from the QUOVADIS Study. Eat Weight Disord. 2012;7(4):e314-e319.

36. Puhl RM, Peterson JL, Luedicke J. Parental perceptions of weight terminology that providers use with youth. Pediatrics. 2011;128(4):e786-e793.

37. Kalarchian MA, Levine MD, Klem ML, Burke LE, Soulakova JN, Marcus MD. Impact of addressing reasons for weight loss on behavioral weight-control outcome. Am J Prev Med. 2011;40(1):18-24.

38. Cresci B, Castellini G, Pala L, Ravaldi C, Faravelli C, Rotella CM, Ricca V. Motivational readiness for treatment in weight control programs: the TREatment MOtivation and REadiness (TRE-MORE) test. J Endocrinol Invest. 2011;34(3):e70-e77.

39. Edmiston FG, Wagner DR. Comparison of methods for setting weight loss goals in males. Fam Med. 2010;42(8):575-576.

40. Jay M, Gillespie C, Schlair S, Sherman S, Kalet A. Physicians' use of the 5As in counseling obese patients: is the quality of counseling associated with patients' motivation and intention to lose weight?. BMC Health Serv Res. 2010;10(1):159.

41. Elfhag K, Rössner S. Initial weight loss is the best predictor for success in obesity treatment and sociodemographic liabilities increase risk for drop-out. Patient Educ Couns. 2010;79(3):361-366.

42. Dalle Grave R, Calugi S, Corica F, Di Domizio S, Marchesini G, QUOVADIS Study Group. Psychological variables associated with weight loss in obese patients seeking treatment at medical centers. J Acad Nutr Diet. 2009;109(12):2010-2016.

43. Cresci B, Rotella CM. Motivational readiness to change in lifestyle modification programs. Eat Weight Disord2009.14(2-3):e158-e162.

44. Kwan S. Competing motivational discourses for weight loss: Means to ends and the nexus of beauty and health. Qual Health Res. 2009;19(9):1223-1233.

45. Chałabis K, Lange E. Self-acceptance motivation and expectations for loosing weight in obese women. Rocz Panstw Zakl Hig. 2008;59(3):351-359.

46. Johannessen KB, Berntsen D. Motivation for weight loss affects recall from autobiographical memory in dieters. Memory. 2009;17(1):69-83.

47. Alm M, Soroudi N, Wylie-Rosett J, Isasi CR, Suchday S, Rieder J, Khan U. A qualitative assessment of barriers and facilitators to achieving behavior goals among obese inner-city adolescents in a weight management program. Diabetes Educ. 2008;34(2):277-284.

48. Heading G. Rural obesity, healthy weight and perceptions of risk: Struggles, strategies and motivation for change. Aust J Rural Health. 2008;16(2):86-91.

49. Annunziato RA, Lowe MR. Taking action to lose weight: toward an understanding of individual differences. Eat Behav. 2007;8(2):185-194.

50. Georgiadis MM, Biddle SJ, Stavrou NA. Motivation for weight-loss diets: A clustering, longitudinal field study using self-esteem and self-determination theory perspectives. Health Educ J. 2006;65(1):53-72.

51. Murtagh J, Dixey R, Rudolf M. A qualitative investigation into the levers and barriers to weight loss in children: opinions of obese children. Arch Dis Child. 2006;91(11):920-923.

52. Dalle Grave R, Calugi S, Molinari E, Petroni ML, Bondi M, Compare A, et al. Weight loss expectations in obese patients and treatment attrition: an observational multicenter study. Obes Res. 2005;13(11):1961-1969.

53. Wannamethee SG, Shaper AG, Lennon L. Reasons for intentional weight loss, unintentional weight loss, and mortality in older men. Arch Intern Med. 2005;165(9):1035-1040.

54. Dalle Grave R, Calugi S, Magri F, Cuzzolaro M, Dall'Aglio E, Lucchin L, et al. Weight loss expectations in obese patients seeking treatment at medical centers. Obes Res. 2004;12(12):2005-2012.

55. Putterman E, Linden W. Appearance versus health: Does the reason for dieting affect dieting behavior?. J Behav Med. 2004;27(2):185-204.

56. Hawks SR, Madanat HN, Merrill RM, Goudy MB, Miyagawa T. A cross-cultural analysis of ‘motivation for eating’as a potential factor in the emergence of global obesity: Japan and the United States. Health Promot Int. 2003;18(2):153-162.

57. Clarke LH. Older women's perceptions of ideal body weights: the tensions between health and appearance motivations for weight loss. Ageing Society. 2002;22(6):751-773.

58. Reeder BA, Chen Y, Macdonald SM, Angel A, Sweet L. Regional and rural-urban differences in obesity in Canada. Canadian Heart Health Surveys Research Group. CMAJ. 1997;157:S10-6.

59. Tinker JE, Tucker JA. Motivations for weight loss and behavior change strategies associated with natural recovery from obesity. Psychol Addict Behav. 1997;11(2):98.

60. Fontaine KR, Cheskin LJ. Predicting treatment attendance and weight loss: assessing the psychometric properties and predictive validity of the Dieting Readiness Test. J Pers Assess. 1997;68(1):173-183.

61. Striegel‐Moore RH, Wilfley DE, Caldwell MB, Needham ML, Brownell KD. Weight‐related attitudes and behaviors of women who diet to lose weight: A comparison of black dieters and white dieters. Obes Res. 1996;4(2):109-116.

62. Harris MB, Waschull S, Walters L. Feeling fat: motivations, knowledge, and attitudes of overweight women and men. Psychol Rep. 1990;67(3_suppl):1191-1202.
